# Durability of immune response after SARS-CoV-2 vaccination in patients with chronic liver disease

**DOI:** 10.3389/fimmu.2023.1200198

**Published:** 2023-06-15

**Authors:** Ruixin Song, Chao Yang, Qianqian Li, Jiayin Wang, Jing Chen, Kai Sun, Hongmin Lv, Yankai Yang, Jing Liang, Qing Ye, YanYing Gao, Jun Li, Ying Li, Junqing Yan, Ying Liu, Tao Wang, Changen Liu, Ping Zhu, Fei Wang, Weili Yin, Huiling Xiang

**Affiliations:** ^1^ The Third Central Clinical College of Tianjin Medical University, Department of Gastroenterology and Hepatology, Tianjin Third Central Hospital, Tianjin Key Laboratory of Extracorporeal Life Support for Critical Diseases, Artificial Cell Engineering Technology Research Center, Tianjin Institute of Hepatobiliary Disease, Tianjin, China; ^2^ Department of Gastroenterology and Hepatology, Tianjin Third Central Hospital, Tianjin Key Laboratory of Extracorporeal Life Support for Critical Diseases, Institute of Hepatobiliary Disease, Tianjin, China; ^3^ Emergency Department, Tianjin Hongqiao Hospital, Tianjin, China

**Keywords:** durability, immune response, SARS-CoV-2, vaccination, chronic liver disease

## Abstract

**Aim:**

The present study aimed to evaluate the durability of immune response after basic and booster immunization with severe acute respiratory syndrome coronavirus 2 (SARS-CoV-2) vaccines in patients with chronic liver disease (CLD).

**Methods:**

Patients with CLD and complete basic or booster immunization with SARS-CoV-2 vaccines were included in this study. Based on the vaccination situation, they were divided into the basic immunity group (Basic) and the booster immunity group (Booster), which were then subdivided into four groups according to the time interval from completion of basic immunization or booster immunization to serological specimen collection. The positive rates and antibody titers of novel coronavirus neutralizing antibody (nCoV NTAb) and novel coronavirus spike receptor-binding domain antibody (nCoV S-RBD) were analyzed.

**Results:**

A total of 313 patients with CLD were enrolled in this study, including 201 in Basic and 112 in Booster. The positive rates of nCoV NTAb and nCoV S-RBD within 30 days of completing basic immunization were 80.4% and 84.8%, respectively, but decreased rapidly with the extension of vaccination time, and only 29% and 48.4% of patients with CLD remained positive for nCoV NTAb and nCoV S-RBD, respectively, after 120 days of completing basic immunization. Within 30 days of booster immunization, the positive rates of nCoV NTAb and nCoV S-RBD in patients with CLD rapidly increased from 29.0% and 48.4% at the end of basic immunization to 95.2% and 90.5%, and maintained a high level (defined as the positive rate >50%) until 120 days when the positive rates of nCoV NTAb and nCoV S-RBD were still high at 79.5% and 87.2%, respectively. After basic immunization, the time for nCoV NTAb and nCoV S-RBD to turn negative was 120 and 169 days, respectively, and the negative time of nCoV NTAb and nCoV S-RBD was significantly prolonged to 266 days and 329 days, respectively.

**Conclusion:**

It is safe and effective for patients with CLD to complete basic and booster immunization with SARS-CoV-2 vaccines. After booster immunization, the immune response of patients with CLD was further improved and the durability of the SARS-CoV-2 antibody was significantly prolonged.

## 1 Introduction

Coronavirus disease-2019 (COVID-19) caused by severe acute respiratory syndrome coronavirus 2 (SARS-CoV-2) is still a major infectious disease endangering human health. Vaccines against SARS-CoV-2 are an effective tool to interrupt the spread of the virus. However, the effectiveness of the vaccine decreases over time. In healthy individuals, the effectiveness of BNT162b2 vaccines in preventing the onset of COVID-19 was 92% between 15 and 30 days after vaccination, which reduced to 47% between 121 and 180 days and was only 23% at 211 days (1). In patients with solid tumors immunized with BNT162b2 vaccines, 15% of novel coronavirus spike receptor-binding domain antibody (nCoV S-RBD)-positive patients turned negative after 6 months (2).

A large number of studies have shown that patients with chronic liver disease (CLD) who received SARS-CoV-2 basic and booster immunization could produce an immune response, but the overall response rate was lower than in healthy people (3–6). Currently, only a few studies have assessed the antibody decay pattern among patients with CLD receiving basic and booster immunization with SARS-CoV-2 vaccines. Thus, the present study aimed to elucidate the dynamic changes in the antibody-positive rate and antibody titer after completing the basic and booster immunization in patients with CLD to clarify the decay pattern of the immune response after SARS-CoV-2 vaccines in patients with CLD. This understanding would provide a basis for the formulation of vaccination strategies during the period of protection against COVID-19 after SARS-CoV-2 vaccination.

## 2 Study design and methods

### 2.1 Participants and subgroups

Outpatients and inpatients who visited the Department of Hepatology, Tianjin Third Central Hospital in China, between January 2021 and June 2022, were enrolled in this study. The inclusion criteria were as follows: 1. Confirmed chronic liver disease; 2. Completion of basic and/or booster immunization of SARS-CoV-2 vaccination; 3. Willingness to follow the study procedure and signed written informed consent.

Group criteria: The patients were grouped according to the dose of SARS-CoV-2 vaccines that had been completed at the time of enrollment. Patients who received only one dose (CanSinoBio Ad5-nCoV vaccine) or two doses (BBIBP-CorV vaccine, CoronaVac vaccine) were classified into the basic immunity group (Basic), and those who completed a booster dose over 6 months after basic immunization comprised the booster immunity group (Booster). Basic and Booster were successively divided into four groups according to the interval from completion of basic immunization or booster immunization to serological specimen collection: ≤30 days, 31–75 days, 76–120 days, and >120 days.

The exclusion criteria were as follows: active or known history of SARS-CoV-2 infection, history of liver transplantation, liver solid tumor, immunosuppression or immunodeficiency status, receiving a systemic immunosuppressant or immunobooster within 3 months prior to screening, and being in a special period (pregnancy and lactation).

### 2.2 Vaccine type and vaccination schedule

Vaccine types were selected according to vaccines available in the community. Four types of vaccines are investigated in this study: BBIBP-CorV vaccine (Beijing Institute of Biological Products Co., Ltd, China), CoronaVac vaccine (Beijing Sinovac Life Sciences Co., Ltd, China), CanSinoBio Ad5-nCoV vaccine (CanSino Biologics Inc. China), and Zifivax vaccine (CHO cell) (Anhui Zhifei Longcom Biopharmaceutical Co., Ltd, China). The vaccination schedule was based on SARS-CoV-2 vaccination guidelines (7). The interval between the first and second doses of SARS-CoV-2 vaccines was 21 days, and the time for receiving the booster dose was >6 months after the previous dose.

### 2.3 Data collection

The data of patients during their first visit to our hospital after basic immunization and/or booster immunization were collected. It included basic information of the participants [age, sex, ethnicity, body mass index (BMI)], liver disease-related information (liver disease etiology, absence or presence of cirrhosis, Child–Pugh classification), COVID-19 vaccine-related information (vaccine type, completion of basic immunization and/or booster immunization time), comorbidity information (hypertension, diabetes, coronary artery disease, arrhythmia), laboratory examination-related indicators [alanine aminotransferase(ALT), aspartate aminotransferase(AST), total bilirubin(TBIL), Platelet(PLT), Creatinine], and the serum to determine novel coronavirus neutralizing antibody (nCoV NTAb) and novel coronavirus spike receptor-binding domain antibody (nCoV S-RBD).

### 2.4 Method for the determination of SARS-CoV-2 antibody

nCoV NTAb and nCoV S-RBD were determined by competitive combination chemiluminescence immunoassay (CLIA) of Mindray Bio-Medical Electronics Co., Ltd (Shenzhen, China). Antibody titer >10.0 AU/mL was considered positive, while that <10.0 AU/mL was considered negative according to the specification.

The operation process consisted of four steps. According to the manufacturer’s protocol, nCoV NTAb and nCoV S-RBD in the serum samples were detected using the particles containing the SARS-CoV-2 antigen. Secondly, the conjugate was added to the antigen-antibody complex, so that the SARS-CoV-2 antigen site could be competitively bound. Thirdly, the unbound reagent was removed by washing, and substrate catalyzed by the conjugate was added to the complex. Finally, nCoV NTAb and nCoV S-RBD in the serum samples were measured by calculating the relative light units of the reactants.

### 2.5 Safety assessment

Data on local and systemic adverse reactions after completing primary and booster immunization with SARS-CoV-2 vaccines were collected in patients with CLD. The local adverse reactions were pain at the injection site, pruritus, swelling, induration, redness, and the severity of various symptoms. Systemic adverse reactions included fatigue, nausea, dizziness, myalgia, fever, anorexia, arthralgia, cough, vomiting, oropharyngeal pain, allergy, dyspnea, syncope, and the severity of various symptoms.

### 2.6 Statistical analysis

The continuous variables were expressed by median and interquartile range, and the Kruskal–Wallis test was used to assess whether the differences between the groups were statistically significant. The categorical variables were expressed by the percentage of patients, and the differences between groups were assessed by χ^2^ tests. Linear regression was used to analyze the change in antibody titer over time in patients with CLD after the SARS-CoV-2 vaccines, and the time for antibody titer to turn negative was calculated. The statistical tests were two-sided tests, and P<0.05 was statistically significant. SPSS 24.0 was used for statistical analysis, and Origin 2022b was employed for mapping.

### 2.7 Ethics

This study was approved by the Ethics Committee of Tianjin Third Central Hospital in China, and the approval number is IRB2021-027-01.

## 3 Results

### 3.1 Study population

**Figure 1:**
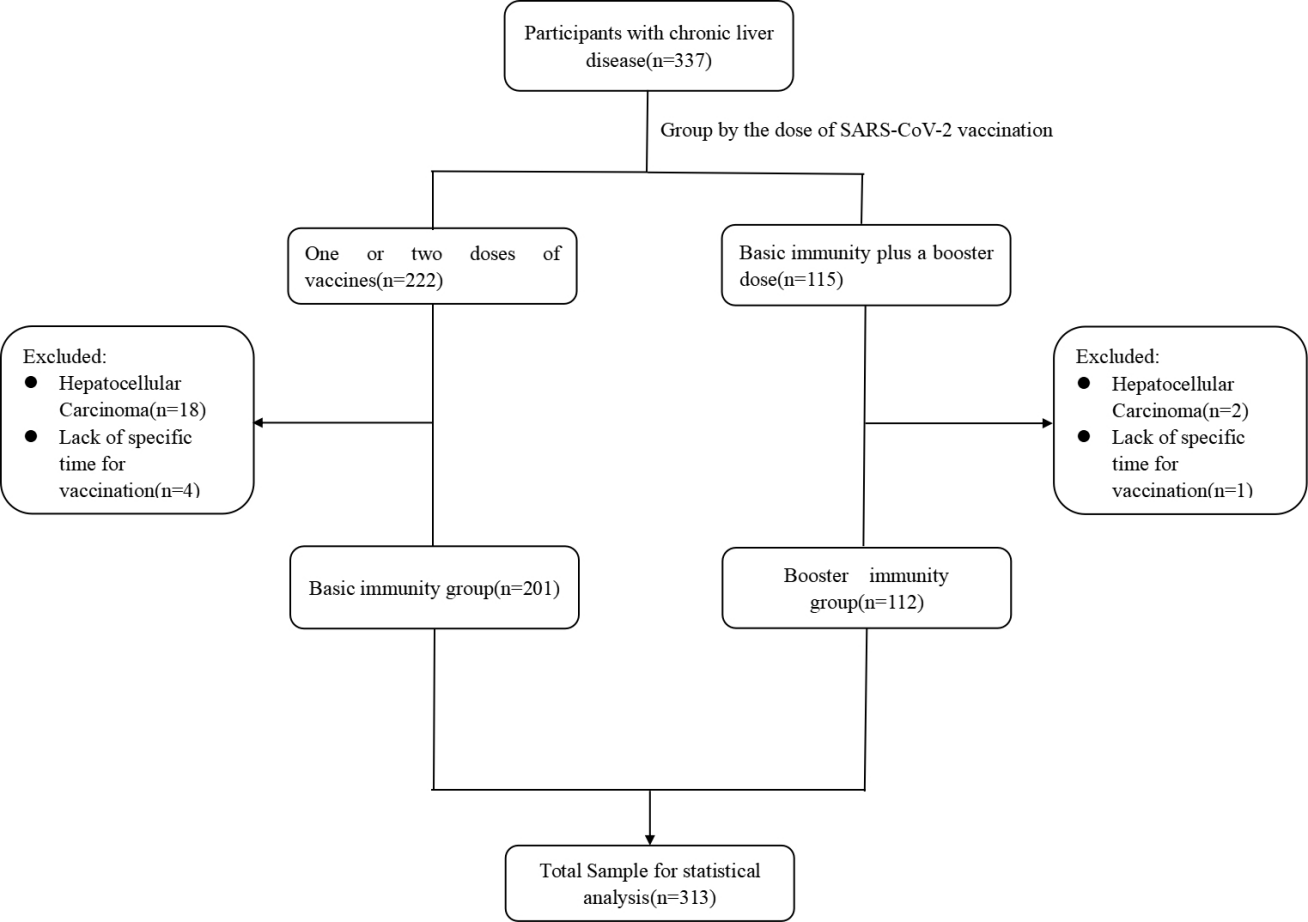
Study flow chart.

A total of 313 patients with CLD were enrolled in this study, including 201 in Basic and 112 in Booster. The median age of the participants was 53.0 [interquartile range (IQR) 42.0–61.0] years, and 58.8% were males. The median BMI was 24.7 (IQR 22.2–27.0) kg/m^2^. Chronic hepatitis B virus, accounting for 74.1% of the 313 patients, was the primary cause of liver disease. Patients with decompensated cirrhosis consisted of 17.2% of the total number of patients with cirrhosis (46.3%). The main events associated with decompensated cirrhosis were ascites (92.0%) and esophagogastric variceal bleeding (16.0%) (**Table 1**; **Figure 1**).

**Table 1:** Demographics, clinical characteristics, and vaccination details in the total analytic sample, the Basic immunity group, and the Booster immunity group at baseline.

Variables	Total	Basic	Booster
N	313	201	112
Age,year	53.0(42.0-61.0)	54.0(41.5-62.0)	52.5(43.3-59.8)
Sex, male	184(58.8)	114(56.7)	70(62.5)
Ethnicity (Han)	306(97.8)	197(98.0)	109(97.3)
BMI	24.7(22.2-27.0)	24.7(22.4-26.8)	24.6(22.1-27.6)
Etiology			
HBV	232(74.1)	135(67.2)	97(86.6)
HCV	23(7.3)	19(9.5)	4(3.6)
ALD	11(3.5)	10(5.0)	1(0.9)
AIH/PBC	16(5.1)	10(5.0)	6(5.4)
others	31(9.9)	27(13.4)	4(3.6)
Presence of cirrhosis	145(46.3)	95(47.3)	50(44.6)
Child-Pugh class			
A	137(94.5)	90(94.7)	47(94.0)
B	4(2.8)	2(2.1)	2(4.0)
C	4(2.8)	3(3.2)	1(2.0)
Decompensated cirrhosis	25(17.2)	17(8.5)	8(7.1)
Decompensation event			
EVB	4(16.0)	3(17.6)	1(12.5)
Ascites	23(92.0)	16(94.1)	7(87.5)
Laboratory indicators			
ALT(U/L)	23.0(17.0-33.0)	24.0(17.6-33.0)	22.0(16.0-36.0)
AST(U/L)	23.0(19.0-30.0)	23.0(19.0-29.3)	22.0(18.0-31.0)
TBIL(μmol/L)	15.6(12.4-20.3)	16.1(13.4-20.6)	14.2(11.0-20.0)
PLT(*10^9^/L)	163.0(124.0-207.0)	148.5(79.3-188.0)	166.0(126.0-210.0)
Creatinine(umol/L)	71.0(62.0-78.0)	72.5(61.8-78.5)	70.5(61.8-78.5)
Vaccine category			
BBIBP-CorV	140(44.7)	95(47.3)	45(40.2)
CoronaVac	142(45.4)	91(45.3)	51(45.5)
CansinoBio	26(8.3)	15(7.5)	11(9.8)
CHO cell Vac	5(1.6)	0(0.0)	5(4.5)
Comorbidities	116(37.1)	78(38.8)	38(33.9)
Hypertension	68(21.7)	48(23.9)	20(17.9)
Diabetes	42(13.4)	30(14.9)	12(10.7)
CAD	9(2.9)	4(2.0)	5(4.5)
Arrhythmia	2(0.6)	1(0.5)	1(0.9)

Data were expressed using median(interquartile range) or frequencies(percentage). The vaccine category refers to the type of first dose SARS-CoV-2 vaccine. BMI, Body Mass Index; HBV, Hepatitis B virus; HCV, Hepatitis C virus; ALD, Alcoholic liver disease; AIH, Autoimmune hepatitis; PBC, Primary biliary cholangitis; EVB, Esophagogastric variceal bleeding; ALT, Alanine aminotransferase; AST, Aspartate aminotransferase; TBIL, Total bilirubin; PLT, Platelet; CAD, Coronary artery disease.

The median age in Basic was 54.0 (IQR 41.5–62.0 years), and no significant difference was detected in the age between the four groups (P=0.107). The proportion of males in the four groups was 73.9%, 53.8%, 41.3%, and 61.3%, respectively (P=0.015). The proportion of patients with hepatitis B virus was 67.2% in Basic (P=0.682). Patients with decompensated cirrhosis consisted of 8.5% of the total number of patients with cirrhosis (47.3%). The main events associated with decompensated cirrhosis were ascites (94.1%) and esophagogastric variceal bleeding (17.6%) (**Table 1**; **Supplement 1**).

The median age in Booster was 52.5 (IQR 43.3–59.8) years and no significant difference was detected in age between the four groups (P=0.118). The proportion of patients with hepatitis B virus was 86.6% in Booster (P=0.191). Patients with decompensated cirrhosis consisted of 7.1% of the total number of patients with cirrhosis (44.6%). The main events associated with decompensated cirrhosis were ascites (87.5%) and esophagogastric variceal bleeding (12.5%) (**Table 1**; **Supplement 2**).

### 3.2 Decay pattern of antibody after SARS-CoV-2 vaccines

#### 3.2.1 Dynamic changes in antibody positivity and titer in basic

After basic immunization of SARS-CoV-2 vaccines, the overall positive rates of nCoV NTAb and nCoV S-RBD were 60.2% and 70.1%, respectively. The median antibody titers were 11.6 (IQR 8.1–20.4) AU/mL and 22.1 (IQR 7.8–64.6) AU/mL, respectively (**Table 2**).

**Table 2:** The dynamic changes of nCoV NTAb and nCoV S-RBD in the Basic immunity group and the Booster immunity group.

	All	≤30	31-75	76-120	>120	P-Value
Basic immunity group						
nCoV NTAb(%)	121(60.2)	37(80.4)	55(70.5)	20(43.5)	9(29.0)	<0.001
nCoV S-RBD(%)	141(70.1)	39(84.8)	57(73.1)	30(65.2)	15(48.4)	0.005
Titer of nCoV NTAb	11.6(8.1-20.4)	18.2(11.2-27.0)	13.3(9.6-25.6)	9.3(6.8-12.9)	8.1(7.4-11.1)	<0.001
Titer of nCoV S-RBD	22.1(7.8-64.6)	43.4(14.2-115.2)	23.6(8.5-69.0)	18.4(7.4-39.3)	8.4(4.2-22.6)	<0.001
Booster immunity group						
nCoV NTAb(%)	98(87.5)	20(95.2)	15(93.8)	32(88.9)	31(79.5)	0.301
nCoV S-RBD(%)	102(91.1)	19(90.5)	15(93.8)	34(94.4)	34(87.2)	0.734
Titer of nCoV NTAb	31.4(15.4-94.1)	69.5(14.3-149.0)	50.7(31.8-157.6)	39.2(20.7-105.2)	19.3(10.9-26.8)	<0.001
Titer of nCoV S-RBD	110.4(30.2-283.7)	289.5(39.5-474.0)	186.6(113.0-339.6)	115.0(49.7-249.2)	54.1(16.8-110.3)	<0.001

They were divided into four groups according to the time(days) between completion of basic immunization or booster immunization and serological specimen collection. Data were expressed using median (interquartile range) or frequencies(percentage). nCoV NTAb(%) represented the positive rate of nCoV NTAb and nCoV S-RBD(%) represented the positive rate of nCoV S-RBD. Antibody titer above 10.0 AU/mL was considered positive, while antibody titer below 10.0 AU/mL was considered negative. nCoV NTAb, novel coronavirus neutralizing antibody; nCoV S-RBD, novel coronavirus spike receptor-binding domain antibody.

**Figure 2:**
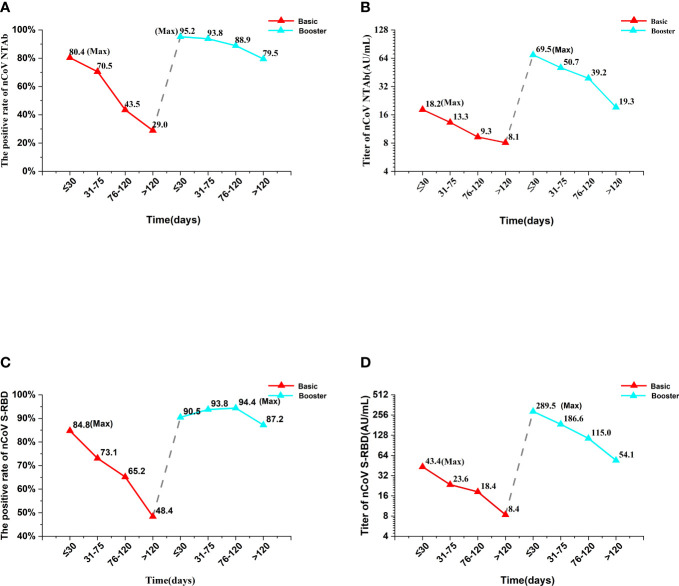
The changes in antibody kinetics in the Basic immunity group and the Booster immunity group. “Max” represented the maximum value of the Basic or Booster immunity group. The red line represented the Basic immunity group and the blue line represented the booster immunity group. Antibody titer above 10.0 AU/mL was considered positive, while antibody titer below 10.0 AU/mL was considered negative. nCoV NTAb, novel coronavirus neutralizing antibody; nCoV S-RBD, novel coronavirus spike receptor-binding domain antibody.

The positive rates of nCoV NTAb and nCoV S-RBD within 30 days of completing basic immunization were 80.4% and 84.8%, respectively, but decreased rapidly with the extension of vaccination time, and only 29.0% and 48.4% of patients with CLD remained positive for nCoV NTAb and nCoV S-RBD after 120 days of completing basic immunization (**Table 2**; **Figures 2A, C**). The titer of nCoV NTAb was 18.2 (IQR 11.2-27.0) AU/mL, 13.3 (IQR 9.6-25.6) AU/mL, 9.3 (IQR 6.8-12.9) AU/mL, and 8.1 (IQR 7.4-11.1) AU/mL within 30 days, 31-75 days, 76-120 days, and 120 days after basic immunization, respectively. The titer of nCoV NTAb peaked at 18.2 (IQR 11.2–27.0) AU/mL within 30 days after basic immunization, and then decayed rapidly in the following three periods, decreasing 26.9%, 48.9%, and 55.5%, respectively. Meanwhile, the titer of nCoV NTAb was only 44.5% of the peak titer after 120 days. The titer of nCoV S-RBD attenuated in a similar pattern. The antibody titer was the highest within 30 days after basic immunization with 43.4 (IQR 14.2–115.2) AU/mL and decreased to 8.4 (IQR 4.2–22.6) AU/mL after 120 days. However, the titer of nCoV S-RBD was 19.4% of the peak titer after 120 days (**Table 2**; **Figures 2B, D**).

#### 3.2.2 Dynamic change in antibody positivity and titer in booster

After booster immunization of SARS-CoV-2 vaccines, the overall positive rates of nCoV NTAb and nCoV S-RBD were 87.5% and 91.1%, respectively. The median antibody titers were 31.4 (IQR 15.4–94.1) AU/mL and 110.4 (IQR 30.2–283.7) AU/mL, respectively, which were significantly higher than those in Basic (**Table 2**).

Within 30 days of booster immunization, the positive rates of nCoV NTAb and nCoV S-RBD in patients with CLD rapidly increased from 29.0% and 48.4% at the end of basic immunization to 95.2% and 90.5%. Notably, the positive rate of nCoV S-RBD lasted longer than nCoV NTAb (**Table 2**; **Figures 2A, C**). Similarly, the antibody titers of nCoV NTAb and nCoV S-RBD in patients with CLD increased rapidly from 8.1 (IQR 7.4–11.1) AU/mL and 8.4 (IQR 4.2–22.6) AU/mL at the end of basic immunization to 69.5 (IQR 14.3–149.0) AU/mL and 289.5 (IQR 39.5–474.0) AU/mL within 30 days after booster immunization, indicating an 8.58- and 34.46-fold increase, respectively. The titer of nCoV S-RBD was significantly higher than that of nCoV NTAb (**Table 2**; **Figures 2B, D**).

After booster immunization, the positive rates of nCoV NTAb and nCoV S-RBD decreased slowly and maintained a high level (defined as a positive rate higher than 50%) until 120 days when the positive rates were still as high as 79.5% and 87.2%, respectively (**Table 2**; **Figures 2A, C**). The titer of nCoV NTAb was 69.5 (IQR 14.3-149.0) AU/mL, 50.7 (IQR 31.8-157.6) AU/mL, 39.2 (IQR 20.7-105.2) AU/mL and 19.3 (IQR 10.9-26.8) AU/mL within 30 days, 31-75 days, 76-120 days, and 120 days after booster immunization, respectively. The titer of nCoV NTAb peaked at 69.5 (IQR 14.3-149.0) AU/mL within 30 days after booster immunization, and then decreased rapidly in the following three periods, 27.1%, 43.6%, and 72.2%, respectively. Meanwhile, the titer of nCoV NTAb was only 27.8% of the peak titer 120 days after booster immunization. The titer of nCoV S-RBD attenuated similarly. The antibody titer was the highest within 30 days after booster immunization with 289.5 (IQR 39.5-474.0) AU/mL and decreased to 54.1 (IQR 16.8-110.3) AU/mL after 120 days. However, the titer of nCoV S-RBD was 18.7% of the peak titer 120 days after booster immunization (**Table 2**; **Figures 2B, D**). In conclusion, the positive rates of nCoV NTAb and nCoV S-RBD did not decrease significantly after the completion of booster immunization, but the titers declined rapidly.

### 3.3 Durability of immune response after basic and booster immunization among patients with CLD

**Figure 3:**
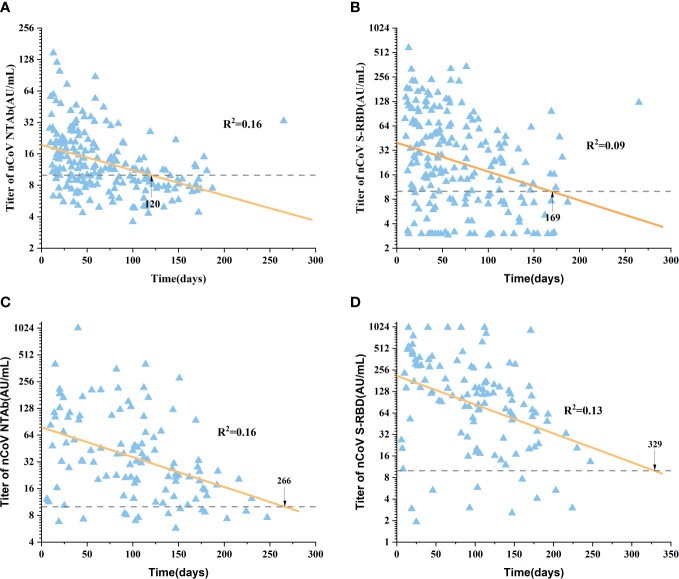
Duration of SARS-CoV-2 antibody in the Basic immunity group and the Booster immunity group. Figures **(A, B)** show the duration of nCoV NTAb and nCoV S-RBD in the Basic immunity group, respectively. Figures **(C, D)** show the duration of nCoV NTAb and nCoV S-RBD in the Booster immunity group, respectively. The dotted line represented the boundary(10.0 AU/mL). nCoV NTAb, novel coronavirus neutralizing antibody; nCoV S-RBD, novel coronavirus spike receptor-binding domain antibody.

Linear regression analysis of antibody titer over time was performed in patients with CLD after basic and booster immunization. The time for nCoV NTAb and nCoV S-RBD to turn negative was 120 and 169 days, respectively (**Figures 3A, B** **)**. Strikingly, after a booster dose, the negative time of nCoV NTAb and nCoV S-RBD was prolonged markedly to 266 and 329 days, respectively (**Figures 3C, D**).

### 3.4 Safety analysis

Adverse reactions to complete basic and booster immunization with SARS-CoV-2 vaccines were mild, appearing as Grade 1 and Grade 2. There were no decompensated events with compensated cirrhosis. In Basic and Booster, the most common local adverse reaction was pain at the injection site, and the most common systemic adverse reaction was fatigue (**Table 3**). There were no significant changes in ALT, AST, TBIL, PLT, and Creatine during follow-up (**Supplement 3**).

**Table 3:** Adverse reactions after basic immunization and booster immunization.

	Basic	Booster
Any adverse reaction		
Grade 1	43(21.4)	29(25.9)
Grade 2	1(0.5)	0(0.0)
Local adverse reaction		
Pain at injection site	17(8.5)	16(14.3)
Pruritus	2(1.0)	1(0.9)
Swelling	1(0.5)	0(0.0)
Induration	0(0.0)	0(0.0)
Redness	0(0.0)	0(0.0)
Systemic adverse reaction		
Fatigue	13(6.5)	5(4.5)
Nausea	4(2.0)	4(3.6)
Dizziness	4(2.0)	3(2.7)
Myalgia	4(2.0)	1(0.9)
Fever	3(1.5)	1(0.9)
anorexia	3(1.5)	2(1.8)
Arthralgia	2(1.0)	1(0.9)
Cough	1(0.5)	1(0.9)
vomiting	0(0.0)	0(0.0)
Oropharyngeal pain	0(0.0)	0(0.0)
Allergy	0(0.0)	0(0.0)
Dyspnea	0(0.0)	0(0.0)
syncope	0(0.0)	0(0.0)

Data were expressed using frequencies (percentage).

## 4 Discussion

COVID-19 has been a major infectious disease threatening human health since SARS-CoV-2 was first reported in 2019. Although SARS-CoV-2 vaccination is an effective method to interrupt transmission (8, 9), the effectiveness of the vaccine decreases over time (10, 11). The inhibition rate of nCoV NTAb in healthy individuals administered with two doses of SARS-CoV-2 vaccines within 6-9 months was 78.0% and had decreased by 17.7% compared to 1-3 months after the vaccination (12). The positive rate of nCoV NTAb was 77.9% on day 56 after two doses of inactivated vaccines, which decreased to 13.2% on day 210. The initial positive rate of nCoV S-RBD was high, closing to 100%, and only 25.0% remained on day 210 (13). The vaccination with the third dose of SARS-CoV-2 vaccines could significantly improve the antibody positivity rate and antibody titer among healthy individuals and the effectiveness of preventing COVID-19 (14, 15). The positive rate of nCoV NTAb peaked at nearly 100% on day 28 after the third dose of SARS-CoV-2 vaccines in healthy people, but the positive rate of antibodies was 68% after 6 months (16). On day 14 of the third dose of inactivated vaccine, the antibody titers of nCoV NTAb and nCoV S-RBD were 711.9 IU/mL and 33.5 S/CO, respectively. After 3 months, nCoV NTAb and nCoV S-RBD titers decreased to 275.2 IU/mL and 17.5 S/CO, respectively.

The positive rate of nCoV NTAb was 90.3% in healthy people administered two doses of SARS-CoV-2 vaccines (6). After basic immunization, the positive rate of nCoV S-RBD among healthcare workers was 99.6% (17). In healthy people with two doses of vaccines, the positive rate of nCoV S-RBD was 100.0% (18). COVID-19 patients with CLD have a worse prognosis and a higher risk of hospitalization and death (19, 20). Previous studies have shown that patients with CLD who receive SARS-CoV-2 vaccines can produce a better immune response (21, 22). In this study, the overall positive rates of nCoV NTAb and nCoV S-RBD were 60.2% and 70.1%, and the median antibody titers were 11.6 AU/mL and 22.1 AU/mL after basic immunization, respectively. After booster immunization, the overall positive rates of nCoV NTAb and nCoV S-RBD increased to 87.5% and 91.1%, and the median antibody titer increased to 31.4 AU/mL and 110.4 AU/mL, respectively. This finding confirmed that SARS-CoV-2 vaccination in patients with CLD was adequate, but the antibody-positive rate and antibody titer was lower than those in healthy individuals, which was consistent with previous results (6, 17, 18).

The positive rate of nCoV S-RBD in healthy people was 100.0% on day 28 after basic immunization, and decreased to 54.1% on day 132 (23). The titer of nCoV S-RBD was 1086 kU/L and 802 kU/L on day 90 and day 180 after basic immunization, which decreased 38.4% and 54.5%, respectively, compared with 1762kU/L on day 30 (24). Healthy people receiving two doses of the Pfizer‐BioNTech vaccines decreased 55.0% about the titer of nCoV S-RBD on day 147 compared with day 28 (25). Currently, only a few studies are available on the decay pattern of antibodies over time after SARS-CoV-2 vaccination in patients with CLD. These results showed that the positive rates of nCoV NTAb and nCoV S-RBD were 80.4% and 84.8% within 30 days after primary immunization. After 120 days, the positive rate was 29.0% and 48.4%, respectively, which was significantly lower than that of healthy people in the same period (23). In this study, the titer of nCoV S-RBD was 43.4 AU/mL, 23.6 AU/mL, 18.4 AU/mL, and 8.4 AU/mL within 30 days, 31-75 days, 76-120 days, and after 120 days after basic immunization, respectively. The titer of nCoV S-RBD peaked at 43.4 AU/mL within 30 days after basic immunization, and then declined rapidly in the following three periods, decreasing 45.6%, 57.6%, and 80.6%, respectively, which were faster than in healthy people (24, 25).

A booster dose of SARS-CoV-2 vaccines could rapidly increase the positive rates of nCoV NTAb and nCoV S-RBD in patients with CLD to 95.2% and 90.5% within 30 days, and maintain a high positive rate for >120 days. Similarly, the antibody titers of nCoV NTAb and nCoV S-RBD in patients with CLD rapidly increased from 8.1 AU/mL and 8.4 AU/mL at the end of basic immunization to 69.5 AU/mL and 289.5 AU/mL within 30 days after booster immunization, indicating that it increased 8.58 times and 34.46 times, respectively. However, the antibody titer decreased rapidly. After the third dose of vaccines, the titer of nCoV S-RBD was 563.0 AU/mL on day 21. After three months, the titer of nCoV S-RBD decreased to 254.5 AU/mL. nCoV S-RBD decreased by 54.8% compared with the titer on day 21 (26). nCoV NTAb and nCoV S-RBD titers decreased to 39.2 AU/mL and 115.0 AU/mL between 76 and 120 days after booster immunization. And the titers of nCoV NTAb and nCoV S-RBD decreased by 43.6% and 60.3% during 76-120 days compared with the peak titer within 30 days, which was significantly faster than the antibody decrease rate in healthy people in the same period (26). After the completion of basic and booster immunization, the peak time of the positive rate and titer in patients with CLD was similar to that in healthy people (26), but the antibody decrease rate at the corresponding time point was slightly faster. The liver produces immune tolerance. In patients with CLD, the liver is in an immunosuppressive microenvironment, which enables adaptive immune cells to develop tolerance, leading to peripheral T cell failure (27).

The half-lives of nCoV NTAb and nCoV S-RBD were 56.26 days and 82.91 days in healthy people after three doses of vaccines, and the antibody titer remained at a high level 3 months after the completion of booster immunization (26). In healthy individuals who received two doses of BNT162b2 vaccines, nCoV NTAb negative time was 252 days (28). In this study, the antibody titer duration in patients with CLD after SARS-CoV-2 vaccines was analyzed further. The results showed that the time of nCoV NTAb and nCoV S-RBD turning negative was 120 and 169 days after receiving basic immunization, respectively. Compared to healthy people, the negative time among patients with CLD was significantly advanced. After a booster dose of the SARS-CoV-2 vaccine, nCoV NTAb and nCoV S-RBD turned negative at 266 and 329 days, respectively. Therefore, it may be necessary to supplement the vaccine 9–12 months after a booster dose in CLD patients.

The spike protein and receptor-binding domain (RBD) are the most reliable antigens for measuring neutralizing antibodies (29). The expression titer of IgM is low, short-lasting, and does not increase with the dose of vaccination (13). IgA plays a major role in protecting the mucosal surface from SARS-CoV-2 infection; however, relevant studies have shown that the titer of IgA induced by SARS-CoV-2 vaccines is not optimal (18), and hence, nCoV NTAb and nCoVS-RBD were determined in this study.

The advantages of this study were as follows: this was the first study to investigate the changes in antibody dynamics, to the best of our knowledge, in patients with CLD after basic and booster immunization. This was helpful because it can reveal the duration of SARS-CoV-2 antibodies and optimize the vaccination strategy. The limitations of this study included the fact that the specimens were from different patients and the study lacked a series of specimens from the same patient. However, the antibody decay pattern obtained was consistent with previous studies, which could explain the persistence of immune response among patients with CLD immunized with SARS-CoV-2 vaccines. The etiology of liver disease is complex and needs to be further classified.

It is safe and effective for patients with CLD to complete basic and booster immunization of SARS-CoV-2 vaccines. After booster immunization, the immune response of patients with CLD was further improved and the durability of SARS-CoV-2 antibodies was significantly prolonged. Therefore, long-term monitoring of the immune response durability to SARS-CoV-2 vaccines is required to optimize vaccination regimens.

## Data availability statement

The raw data supporting the conclusions of this article will be made available by the authors, without undue reservation.

## Ethics statement

This study was approved by the Ethics Committee of Tianjin Third Central Hospital in China, and the approval number is IRB2021-027-01. The patients/participants provided their written informed consent to participate in this study.

## Author contributions

All authors listed have made a substantial, direct, and intellectual contribution to the work and approved it for publication.
