# Supplementary material for: Durability of immune response after SARS-CoV-2 vaccination in patients with chronic liver disease
Source: Front Immunol. 2023 Jun 15;14:1200198. doi: 10.3389/fimmu.2023.1200198 (PMC10308026; doi:10.3389/fimmu.2023.1200198)
Supplement: Supplementary file 2 [file Table_2.docx]

**Supplement 2 Demographics, clinical characteristics, and vaccination details in the Booster immunity group at baseline.**

| **Variables** | **≤30** | **31-75** | **76-120** | **>120** | **P-Value** |
| --- | --- | --- | --- | --- | --- |
| **N** | 21 | 16 | 36 | 39 |  |
| **Age, year** | 57.0(43.5-66.0) | 59.0(41.3-66.0) | 52.0(44.0-59.5) | 51.0(40.0-56.0) | 0.118 |
| **Sex, male** | 13(61.9) | 10(62.5) | 21(58.3) | 26(66.7) | 0.903 |
| **Ethnicity (Han)** | 21(100.0) | 16(100.0) | 36(100.0) | 36(92.3) | 0.691 |
| **BMI** | 24.8(21.8-27.8) | 24.7(22.2-26.5) | 23.7(22.1-27.2) | 25.4(22.3-27.6) | 0.714 |
| **Etiology** |  |  |  |  | 0.191 |
| HBV | 16(76.2) | 14(87.5) | 32(88.9) | 35(89.7) |  |
| HCV | 1(4.8) | 2(12.5) | 1(2.8) | 0(0.0) |  |
| ALD | 1(4.8) | 0(0.0) | 0(0.0) | 0(0.0) |  |
| AIH/PBC | 3(14.3) | 0(0.0) | 1(2.8) | 2(5.1) |  |
| others | 0(0.0) | 0(0.0) | 2(5.6) | 2(5.1) |  |
| **Presence of cirrhosis** | 9(42.9) | 7(43.8) | 17(47.2) | 17(43.6) | 0.985 |
| **Child-Pugh class** |  |  |  |  | 0.843 |
| A | 8(88.9) | 7(100.0) | 16(94.1) | 16(94.1) |  |
| B | 0(0.0) | 0(0.0) | 1(5.9) | 1(5.9) |  |
| C | 1(11.1) | 0(0.0) | 0(0.0) | 0(0.0) |  |
| **Decompensated cirrhosis** | 2(22.2) | 1(14.3) | 1(5.9) | 4(23.5) | 0.672 |
| **Decompensation event** |  |  |  |  | 0.675 |
| EVB | 0(0.0) | 0(0.0) | 0(0.0) | 1(25.0) |  |
| Ascites | 2(100.0) | 1(100.0) | 1(100.0) | 3(75.0) |  |
| **Laboratory indicators** |  |  |  |  |  |
| ALT(U/L) | 19.0(16.0-43.0) | 23.0(12.0-42.5) | 25.0(18.3-34.8) | 18.0(15.0-35.0) | 0.561 |
| AST(U/L） | 24.0(18.5-39.0) | 24.0(17.3-27.8) | 24.0(19.0-30.0) | 20.0(17.0-24.8) | 0.466 |
| TBIL(μmol/L) | 15.3(10.0-20.8) | 13.5(9.2-17.6) | 14.1(12.1-20.8) | 14.1(11.0-22.6) | 0.826 |
| PLT(*10^9^/L) | 151.0(113.8-193.0) | 179.0(136.0-203.0) | 172.0(124.0-214.0) | 179.0(125.0-227.0) | 0.525 |
| Creatinine(umol/L) | 68.5(63.5-79.3) | 75.0(66.0-80.0) | 67.5(56.5-77.8) | 72.0(61.5-79.0) | 0.778 |
| **Vaccine category** |  |  |  |  | 0.789 |
| BBIBP-CorV | 7(33.3) | 4(25.0) | 16(44.4) | 18(46.2) |  |
| CoronaVac | 12(57.1) | 8(50.0) | 15(41.7) | 16(41.0) |  |
| CansinoBio | 1(4.8) | 3(18.8) | 3(8.3) | 4(10.3) |  |
| CHO cell Vac | 1(4.8) | 1(6.3) | 2(5.6) | 1(2.6) |  |
| **Comorbidities** | 5(23.8) | 4(25.0) | 17(47.2) | 12(30.8) | 0.209 |
| Hypertension | 4(19.0) | 3(18.8) | 9(25.0) | 4(10.3) | 0.420 |
| Diabetes | 1(4.8) | 2(12.5) | 3(8.3) | 6(15.4) | 0.604 |
| CAD | 2(9.5) | 0(0.0) | 2(5.6) | 1(2.6) | 0.484 |
| Arrhythmia | 0(0.0) | 1(6.3) | 0(0.0) | 0(0.0) | 0.143 |

They were divided into four groups according to the time(days) between the completion of booster immunization and serological specimen collection. Data were expressed using median(interquartile range) or frequencies(percentage). Vaccine category referred to the type of first dose SARS-CoV-2 vaccine. BMI, Body Mass Index; HBV, Hepatitis B virus; HCV, Hepatitis C virus; ALD, Alcoholic liver disease; AIH, Autoimmune hepatitis; PBC, Primary biliary cholangitis; EVB, Esophagogastric variceal bleeding; ALT, Alanine aminotransferase; AST, Aspartate aminotransferase; TBIL, Total bilirubin; PLT, Platelet; CAD, Coronary artery disease.
