# Supplementary material for: Durability of immune response after SARS-CoV-2 vaccination in patients with chronic liver disease
Source: Front Immunol. 2023 Jun 15;14:1200198. doi: 10.3389/fimmu.2023.1200198 (PMC10308026; doi:10.3389/fimmu.2023.1200198)
Supplement: Supplementary file 3 [file Table_3.docx]

**Supplement 3 The changes of laboratory indicators in the Basic immunity group and the Booster immunity group during follow-up.**

| **Variables** | **Basic** | | | | **Booster** | | | |
| --- | --- | --- | --- | --- | --- | --- | --- | --- |
|  | **≤30** | **31-75** | **76-120** | **>120** | **≤30** | **31-75** | **76-120** | **>120** |
| **ALT(U/L)** | 24.0(17.0-36.0) | 24.5(16.3-38.3) | 24.0(15.0-41.5) | 32.0(18.0-103.0) | 26.0(17.0-36.5) | 23.5(14.0-33.0) | 23.0(16.0-38.5) | 23.0(17.0-34.0) |
| **AST(U/L）** | 23.0(20.0-31.0) | 24.0(19.3-36.3) | 23.0(19.3-40.8) | 24.0(20.0-66.0) | 28.0(19.5-40.0) | 22.0(18.0-27.8) | 21.0(19.0-35.5) | 21.0(19.0-40.0) |
| **TBIL(μmol/L)** | 18.8(12.5-26.4) | 16.3(12.6-22.4) | 17.5(13.8-24.9) | 20.8(13.4-30.9) | 19.0(13.0-24.0) | 13.8(9.2-16.3) | 14.3(10.9-20.5) | 14.7(11.6-19.0) |
| **PLT(*10^9^/L)** | 65.5(44.3-212.8) | 145.0(139.5-211.0) | 143.0(99.5-182.8) | 127.0(76.3-174.0) | 138.0(99.3-177.5) | 197.0(133.5-224.0) | 176.5(154.3-259.3) | 174.0(135.0-224.0) |
| **Creatinine(umol/L)** | 73.0(57.8-77.0) | 70.5(60.3-86.8) | 71.0(59.3-81.3) | 61.5(59.5-69.5) | 69.0(59.0-79.0) | 69.0(63.5-78.5) | 66.0(56.0-81.0) | 70.5(62.0-76.0) |

They were divided into four groups according to the time(days) between completion of basic immunization or booster immunization and serological specimen collection. Data were expressed using the median (interquartile range). ALT, Alanine aminotransferase; AST, Aspartate aminotransferase; TBIL, Total bilirubin; PLT, Platelet.
